# Nothing Made in Vain

**Published:** 1871-09

**Authors:** 


### NOTHING MADE IN VAIN.

New cures, new remedies for disease, new articles of food and clothing are claiming the attention of mankind from time to time, giving us a deeper, clearer, and more convincing proof of the wisdom and benevolence of Him who doeth all things well. Within easy memory of multitudes now living, there was an article in our gardens which was cultivated as a mere matter of ornament; so red, so smooth, so beautiful, as to have the name of " Love Apple," given to it, now everywhere known as

### THE TOMATO,

Which has become one of the most universally used articles of the table, palatable, nutritious, healthful. It would be difficult, perhaps, to find a well-to-do family which did not use it. Whether raw, or stewed, or hot, or cold, or fresh, or preserved, it is alike the favorite of the multitude. Its acidity plays the same healthful, cooling part in the body, as the acidity of fruits and berries; while its seeds act in such a manner as to regulate the system, as do the seeds of the white mustard and the fig.

### SMALL-POX

In its last stages, seems to have been cured by a tea made of the root known as the " Ladies' Saddle " or " Water Cup,"

having the botanical name of *Serracenia purpura.* It is said that at a Massachusetts almshouse, its free use in sixty cases of small-pox was followed by the recovery of all but one. It seems to allay the fever and irritation of the pustules, and dries them up rapidly, leaving scarcely a trace of a mark.

CANCER

Has been considered among the best educated physicians as an incurable disease. Sometimes marked benefit seemed for a time to follow the "cutting out" of the cancer, but it only hastens a fatal result, for generally, sooner or later, it breaks out in several places instead of one. The essence of the disease seems to be a depravation of the blood, of a nature similar to the fundamental cause of consumption, only attacking the flesh, instead of the lungs. Yet within a few months, a plant from South America has had public attention drawn to it, and seems to be efficacious in curing that dreadful malady; dreadful, not only because considered incurable, but from the insufferable noisomeness of the sore, its tedious and unendurable agony, when attacking some parts of the body. So clear has been the proof of the curative power of this newly discovered agent, that a gentleman has been sent, under the auspices of the government, to the distant locality of its growth, with the intention, ultimately, of encouraging its cultivation at home, if as efficacious as claimed.

COTTON SEED,

Many years ago, was considered a nuisance on the "plantation;" various ways were devised of getting rid of it. We have seen immense piles of it burned up. Now, an oil has been made of it, so valuable for many purposes, that it has become an object of mercantile importance. No doubt, in the progress of events, it will be found that there are other plants good for food, for clothing, for the prevention and cure of disease, each one giving new proof of the Scripture declaration; "His loving kindness is over all his works;"

that we " are the sheep of his pasture, and the work of his hands."

